# Supplementary figures and images for: Cerebrovascular reactivity assessment with O2-CO2 exchange ratio under brief breath hold challenge
Source: PLoS One. 2020 Mar 24;15(3):e0225915. doi: 10.1371/journal.pone.0225915 (PMC7092994; doi:10.1371/journal.pone.0225915)

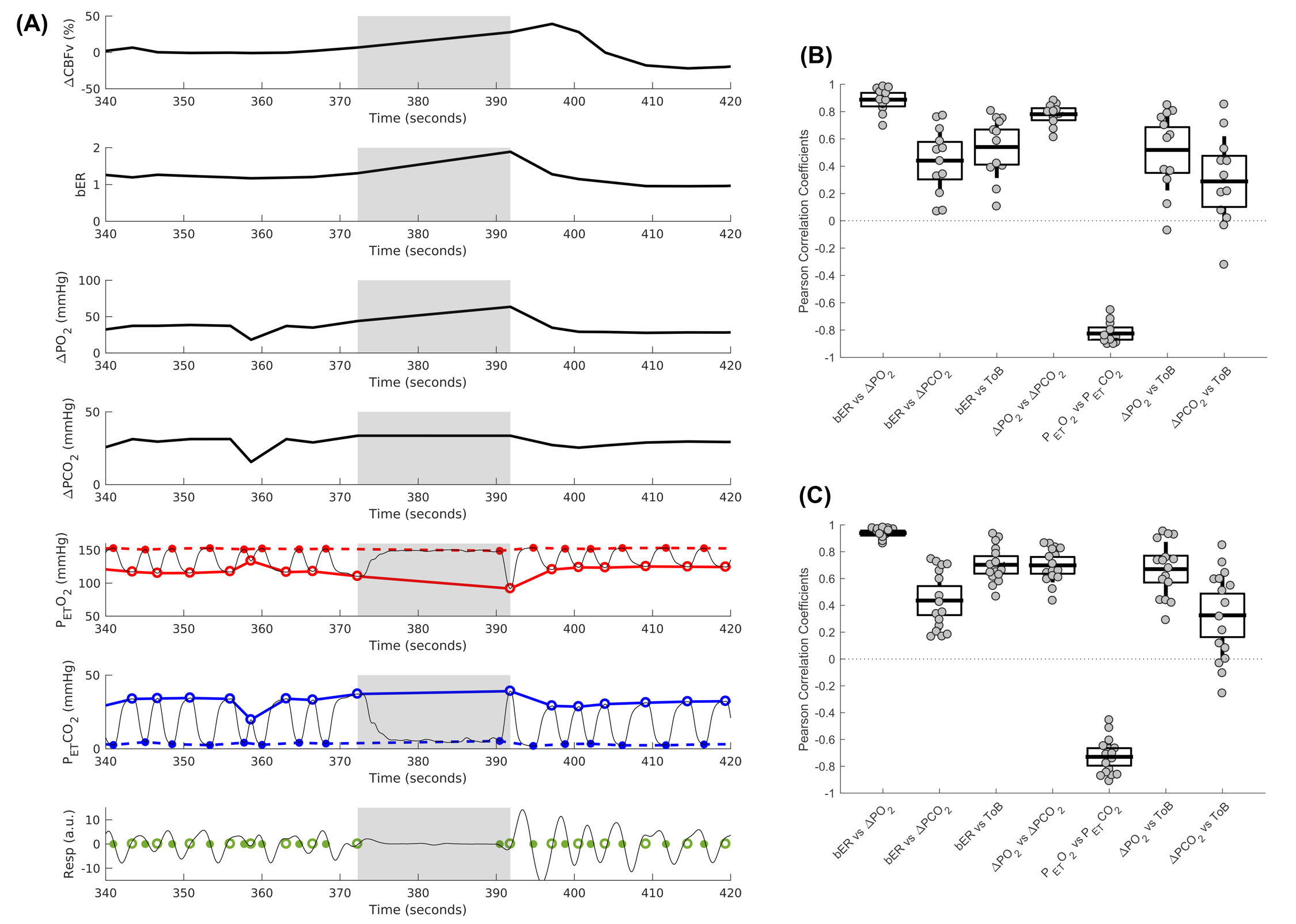

Supplement: S1 Fig — (A) A segment of 80-second time series of ΔCBFv in left MCA and physiological changes including breath-by-breath bER, ΔPO2, ΔPCO2, PETO2 and PETCO2 measured by gas analyzers and respiration time series (Resp) measured by respiratory bellow in a representative subject under breath hold challenge in TCD session. Open circles represent end expiration while closed circles represent end inspiration in resting phase or onset of expiration at the end of breath hold epoch. Positive phases with deflection above zero on the respiration time series represent inspiration and negative phases with deflection below zero represent expiration. The inspiratory and expiratory phases of each respiratory cycle on the time series of PETO2 and PETCO2 are verified by those on respiration time series. The timing for open (end expiration) and closed (end inspiration) circles in green is the same as those in red and blue. (B) Correlations among breath-by breath respiratory matrices (bER, ΔPO2, ΔPCO2, ToB, PETO2 and PETCO2) in all subjects who participated in TCD sessions (n = 12), and (C) those who participated in MRI sessions (n = 16). Each gray circle represents the Pearson’s correlation coefficient from the correlation analysis of the time series of parameter pair shown on x-axis for each subject. The thick middle horizontal line, the box and the vertical rod represent the mean, 95% confidence interval and standard deviation of the group data respectively. The time series of bER had stronger correlation with that of ΔPO2 than ΔPCO2, although both ΔPO2 and ΔPCO2 contributed to changes of bER. The correlation coefficients from ΔPO2 vs ΔPCO2 varied from 0.6 to 0.9 in TCD sessions and from 0.4 to 0.9 in MRI sessions, suggesting that ΔPO2 and ΔPCO2 are not necessarily redundant. The difference in the ranges of correlation strength found between TCD and MRI sessions may be due to the difference in posture of the subjects, where the subjects were in erect seated position in TCD sessions and they [file pone.0225915.s001.tif]

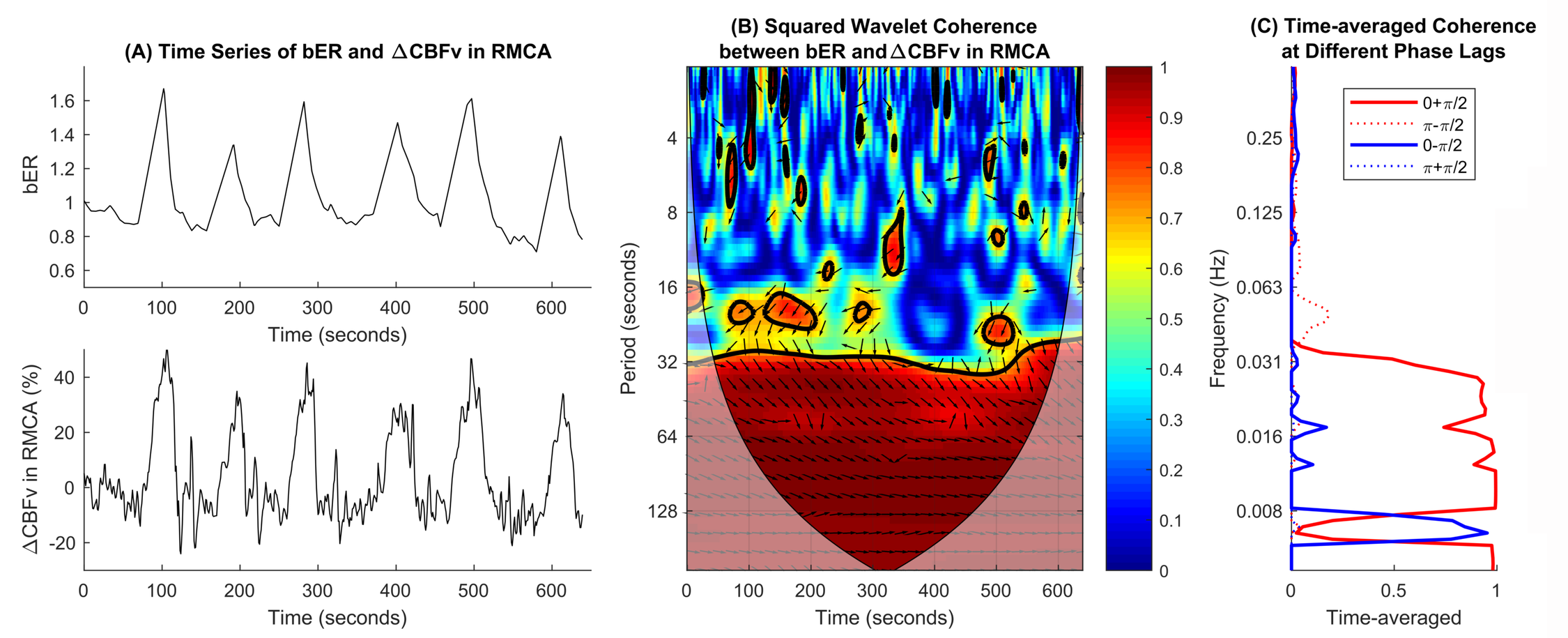

Supplement: S2 Fig — (A) Time series of bER and ΔCBFv measured in right MCA in a representative subject under breath hold challenge. (B) The squared wavelet coherence between these two time series. Squared wavelet coherence is plotted with x-axis as time and y-axis as scale which has been converted to its equivalent Fourier period. The magnitude of wavelet transform coherence ranges between 0 and 1, where warmer color represents stronger coherence and cooler color represents weaker coherence. Areas inside the ‘cone of influence’, which are locations in the time-frequency plane where edge effects give rise to lower confidence in the computed values, are shown in faded color outside of the conical contour. The statistical significance level of the wavelet coherence is estimated using Monte Carlo methods and the 5% significance level against red noise is shown as thick contour. The phase angle between the two time series at particular samples of the time-frequency plane is indicated by an arrow (rightward pointing arrows indicate that the time series are in phase or positively correlation, leftward pointing arrows indicate anticorrelation and the downward pointing arrows indicate phase angles of π/2). There are four different ranges of phase lags: 0+π/2, 0-π/2, π-π/2, and π+π/2. (C) Time-averaged coherences at four different phase lags of 0+π/2, 0-π/2, π-π/2, and π+π/2. At each phase lag range, time-averaged coherence was defined as the total significant coherence at each scale where the wavelet coherence magnitude exceeded 95% significance level, normalized by the maximum possible coherence outside the cone of influence, i.e. inside the conical contour, at that particular scale and phase lag range. (TIF) [file pone.0225915.s002.tif]

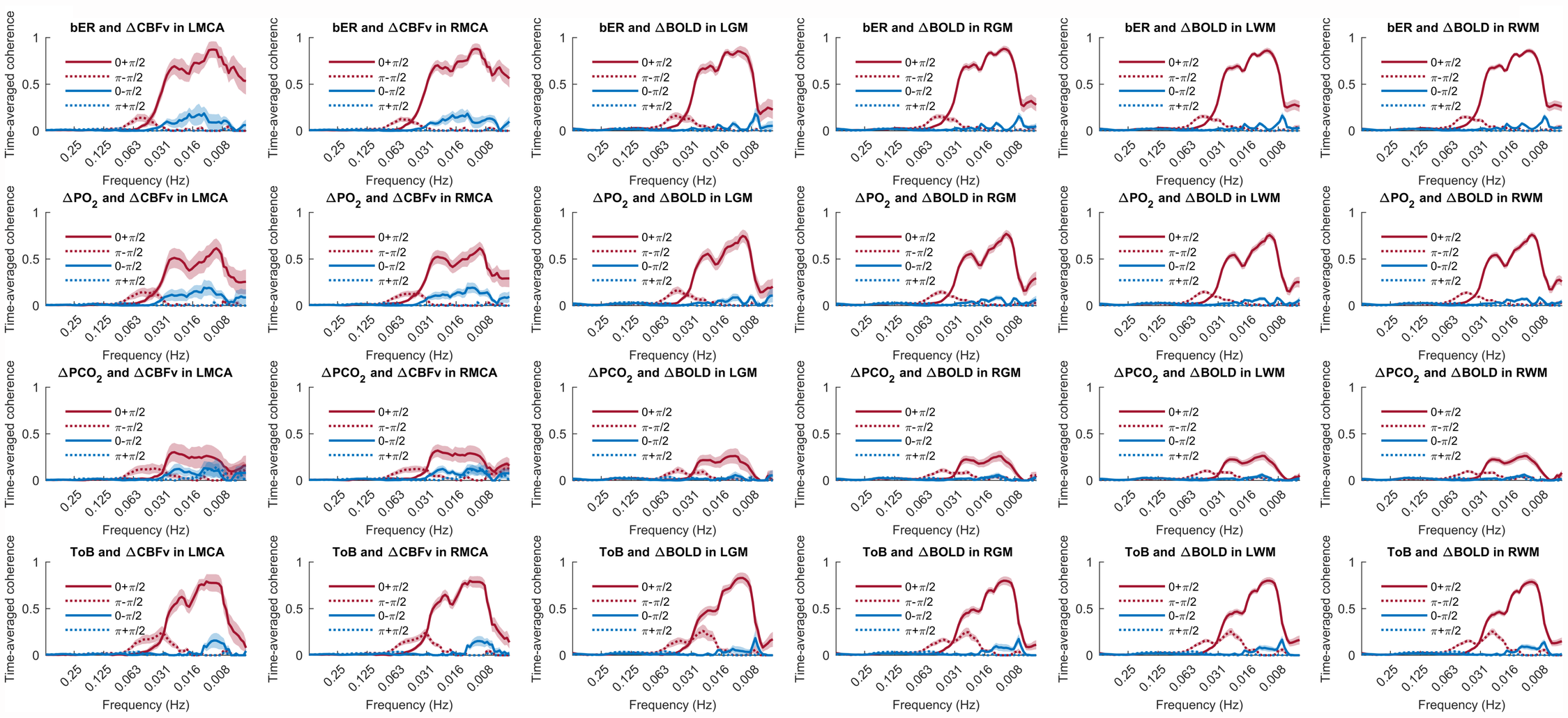

Supplement: S3 Fig — The mean time-averaged coherence between time series of respiratory metrics and cerebral hemodynamic responses (ΔCBFv in LMCA and RMCA, and ΔBOLD in LGM, RGM, LWM and RWM) at four different phase lags (0+π/2, 0-π/2, π-π/2, and π+π/2) for the subjects included in the TCD sessions (n = 12) and in the MRI sessions (n = 16). Color shaded areas represent SEM. Comparing with ΔPO2, ΔPCO2 and ToB, the total time-averaged coherence between bER and cerebral hemodynamic responses was found to be significantly stronger between 0.008Hz (1/128 seconds) and 0.03Hz (1/32 seconds). The strong mean time-averaged coherence between respiratory metrics and cerebral hemodynamic responses were found at phase lag of 0+π/2. (TIF) [file pone.0225915.s003.tif]

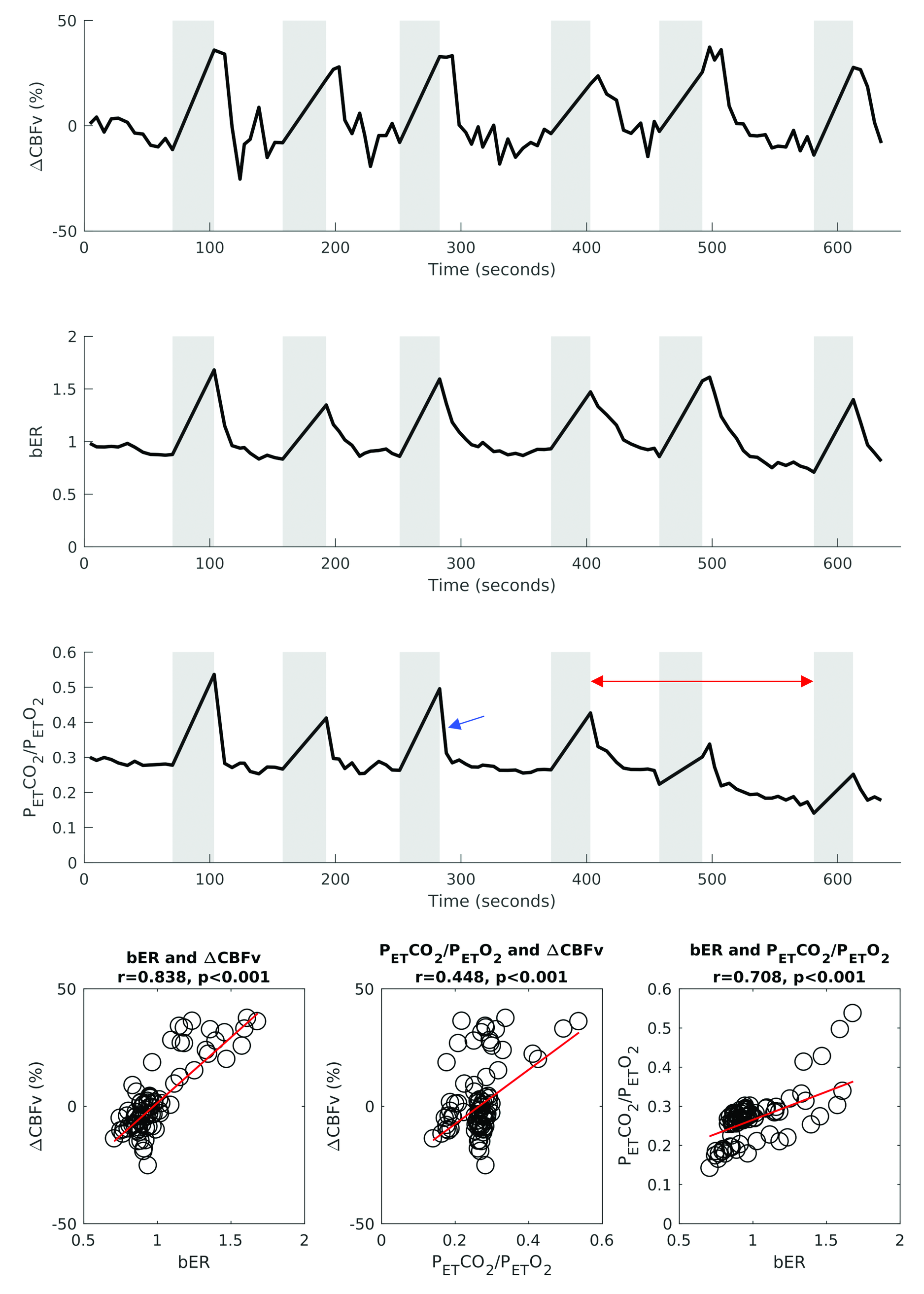

Supplement: S4 Fig — Time series of ΔCBFv in left MCA, bER and PETCO2/PETO2 in the same representative subject in Fig 2A under breath hold challenge in TCD session. Shaded areas represent breath hold periods. The time series of bER followed closely to the ΔCBFv changes, while PETCO2/PETO2 did not follow ΔCBFv changes some time during the challenge as indicated by two-headed arrow in red. In the time period between 400 and 580 seconds (as indicated by two-headed arrow) when the subject had shallow breathing, the amplitude of PETCO2/PETO2 decreased significantly in comparison with that of bER. This may be attributed to the different property of gas measurements where the time series of ΔPO2 and ΔPCO2 oscillated in phase while those of PETO2 and PETCO2 oscillated out of phase (Fig 2A). PETCO2 was decreased and PETO2 was increased by shallow breathing, resulting in a significant decrease in PETCO2/PETO2. (TIF) [file pone.0225915.s004.tif]
