# Supplementary material for: Cerebrovascular reactivity assessment with O2-CO2 exchange ratio under brief breath hold challenge
Source: PLoS One. 2020 Mar 24;15(3):e0225915. doi: 10.1371/journal.pone.0225915 (PMC7092994; doi:10.1371/journal.pone.0225915)
Supplement: S1 Table — Strength of correlation indicated by Pearson’s correlation coefficients among respiratory metrics including bER, ΔPO2, ΔPCO2, ToB, PETO2 and PETCO2 in all subjects who participated in TCD sessions (n = 12), and those who participated in MRI sessions (n = 16). The time series of bER had stronger correlation with that of ΔPO2 than ΔPCO2, although both ΔPO2 and ΔPCO2 contributed to changes of bER. The correlation coefficients from ΔPO2 vs ΔPCO2 varied from 0.6 to 0.9 in TCD sessions and from 0.4 to 0.9 in MRI sessions, suggesting that ΔPO2 than ΔPCO2 are not necessarily redundant. (DOCX) [file pone.0225915.s005.docx]

**S1 Table. Correlation among RGE metrics.**

| **Subjects** | **bER**  **vs**  **ΔPO_2_** | **bER**  **vs**  **ΔPCO_2_** | **bER**  **vs**  **ToB** | **ΔPO_2_**  **vs**  **ΔPCO_2_** | **P_ET_O_2_**  **vs**  **P_ET_CO_2_** | **ΔPO_2_**  **vs**  **ToB** | **ΔPCO_2_**  **vs**  **ToB** |
| --- | --- | --- | --- | --- | --- | --- | --- |
|  | ***TCD sessions*** | | | | | | |
| s4 | 0.777* | 0.326* | 0.389* | 0.840* | -0.878* | 0.374* | 0.207⁑ |
| s5 | 0.888* | 0.341* | 0.420* | 0.730* | -0.799* | 0.365* | 0.076 |
| s6 | 0.916* | 0.520* | 0.665* | 0.799* | -0.840* | 0.700* | 0.439* |
| s7 | 0.969* | 0.672* | 0.653* | 0.826* | -0.870* | 0.628* | 0.332* |
| s8 | 0.944* | 0.583* | 0.725* | 0.811* | -0.849* | 0.756* | 0.526* |
| s9 | 0.883* | 0.438* | 0.105 | 0.802* | -0.849* | 0.122 | 0.019 |
| s10 | 0.934* | 0.532* | 0.805* | 0.783* | -0.902* | 0.788* | 0.437* |
| s11 | 0.984* | 0.759* | 0.753* | 0.857* | -0.892* | 0.805* | 0.713* |
| s12 | 0.977* | 0.771* | 0.751* | 0.881* | -0.902* | 0.847* | 0.852* |
| s14 | 0.696* | 0.068 | 0.229† | 0.757* | -0.719* | -0.070 | -0.322* |
| s15 | 0.855* | 0.203‡ | 0.403* | 0.673* | -0.654* | 0.302* | -0.034 |
| s17 | 0.827* | 0.075 | 0.585* | 0.612* | -0.752* | 0.606* | 0.216‡ |
|  | ***BOLD sessions*** | | | | | | |
| s1 | 0.887* | 0.166 | 0.613* | 0.595* | -0.649* | 0.439* | -0.106 |
| s2 | 0.976* | 0.745* | 0.819* | 0.864* | -0.873* | 0.901* | 0.849* |
| s3 | 0.953* | 0.471* | 0.934* | 0.709* | -0.778* | 0.951* | 0.594* |
| s4 | 0.862* | 0.336* | 0.645* | 0.762* | -0.820* | 0.438* | -0.032 |
| s5 | 0.963* | 0.658* | 0.629* | 0.818* | -0.846* | 0.617* | 0.317⁑ |
| s6 | 0.969* | 0.726* | 0.704* | 0.860* | -0.911* | 0.757* | 0.597* |
| s7 | 0.972* | 0.425* | 0.881* | 0.619* | -0.607* | 0.931* | 0.642* |
| s8 | 0.914* | 0.247‡ | 0.785* | 0.608* | -0.672* | 0.736* | 0.214‡ |
| s9 | 0.975* | 0.700* | 0.722* | 0.833* | -0.867* | 0.734* | 0.509* |
| s10 | 0.980* | 0.706* | 0.907* | 0.825* | -0.863* | 0.928* | 0.720* |
| s11 | 0.880* | 0.204‡ | 0.544* | 0.642* | -0.667* | 0.290* | -0.256⁑ |
| s12 | 0.958* | 0.168 | 0.648* | 0.435* | -0.514* | 0.592* | 0.002 |
| s13 | 0.909* | 0.293⁑ | 0.465* | 0.654* | -0.712* | 0.419* | 0.116 |
| s14 | 0.932* | 0.183 | 0.579* | 0.521* | -0.456* | 0.678* | 0.418* |
| s15 | 0.968* | 0.597* | 0.697* | 0.773* | -0.742* | 0.743* | 0.547* |
| s16 | 0.928* | 0.349* | 0.663* | 0.660* | -0.705* | 0.571* | 0.081 |

**p≤0.001, ⁑p≤0.005, †p≤0.01, ‡p≤0.05*

Strength of correlation indicated by Pearson’s correlation coefficients among respiratory metrics including bER, ΔPO_2_, ΔPCO_2_, ToB, P_ET_O_2_ and P_ET_CO_2_ in all subjects who participated in TCD sessions (n=12), and those who participated in MRI sessions (n=16). The time series of bER had stronger correlation with that of ΔPO_2_ than ΔPCO_2_, although both ΔPO_2_ and ΔPCO_2_ contributed to changes of bER. The correlation coefficients from ΔPO_2_ vs ΔPCO_2_ varied from 0.6 to 0.9 in TCD sessions and from 0.4 to 0.9 in MRI sessions, suggesting that ΔPO_2_ than ΔPCO_2_ are not necessarily redundant.
