# Supplementary material for: Cerebrovascular reactivity assessment with O2-CO2 exchange ratio under brief breath hold challenge
Source: PLoS One. 2020 Mar 24;15(3):e0225915. doi: 10.1371/journal.pone.0225915 (PMC7092994; doi:10.1371/journal.pone.0225915)
Supplement: S2 Table — A. Correlation between RGE metrics and ΔCBFv in TCD sessions. Strength of correlation indicated by Pearson’s correlation coefficients between ΔCBFv and RGE metrics including bER, ΔPO2, ΔPCO2 and ToB (n = 12). Numbers in brackets next to Pearson’s correlation coefficients indicate p values from individual correlation analyses. The bottom row shows the mean values of Fisher Z scores transformed from Pearson’s correlation coefficients in groups. Numbers in brackets next to mean Fisher Z scores indicate p values in the paired comparisons. The correlation between ΔCBFv and bER was significantly larger than those of the correlation between ΔCBFv and the other respiratory metrics in the paired comparisons (p<0.001). bER is the only parameter that consistently showed significantly high correlation with the ΔCBFv measured in LMCA and RMCA. B. Correlation between RGE metrics and ΔBOLD in MRI sessions. Strength of correlation indicated by Pearson’s correlation coefficients between ΔBOLD and RGE metrics including bER, ΔPO2, ΔPCO2 and ToB (n = 16). Numbers in brackets next to Pearson’s correlation coefficients indicate p values from individual correlation analyses. The bottom row shows the mean values of Fisher Z scores transformed from Pearson’s correlation coefficients in groups. Numbers in brackets next to mean Fisher Z scores indicate p values in paired comparisons. The correlation between ΔBOLD and bER was significantly larger than those of the correlation between ΔBOLD and the other respiratory metrics in the paired comparisons (p<0.001). bER is the only parameter that consistently showed significantly high correlation with the ΔBOLD measured in LGM, RGM, LWM and RWM. (DOCX) [file pone.0225915.s006.docx]

**S2 Table A. Correlation between RGE metrics and ∆CBFv in TCD sessions.**

|  | **ΔCBFv in LMCA** | | | | **ΔCBFv in RMCA** | | | |
| --- | --- | --- | --- | --- | --- | --- | --- | --- |
| **Subjects** | **bER** | **ΔPO_2_** | **ΔPCO_2_** | **ToB** | **bER** | **ΔPO_2_** | **ΔPCO_2_** | **ToB** |
| s4 | 0.819 (<0.001) | 0.687 (<0.001) | 0.349 (<0.001) | 0.404 (<0.001) | 0.813 (<0.001) | 0.674 (<0.001) | 0.338 (<0.001) | 0.433 (<0.001) |
| s5 | 0.712 (<0.001) | 0.575 (<0.001) | 0.158 (0.122) | 0.318 (0.002) | 0.813 (<0.001) | 0.668 (<0.001) | 0.172 (0.092) | 0.491 (<0.001) |
| s6 | 0.838 (<0.001) | 0.685 (<0.001) | 0.264 (0.016) | 0.462 (<0.001) | 0.842 (<0.001) | 0.705 (<0.001) | 0.302 (0.006) | 0.460 (<0.001) |
| s7 | 0.754 (<0.001) | 0.645 (<0.001) | 0.302 (0.002) | 0.511 (<0.001) | 0.717 (<0.001) | 0.626 (<0.001) | 0.331 (0.001) | 0.486 (<0.001) |
| s8 | 0.733 (<0.001) | 0.556 (<0.001) | 0.072 (0.399) | 0.410 (<0.001) | 0.719 (<0.001) | 0.548 (<0.001) | 0.072 (0.400) | 0.421 (<0.001) |
| s9 | 0.433 (<0.001) | 0.277 (0.023) | -0.043 (0.730) | 0.349 (0.004) | 0.396 (0.001) | 0.247 (0.044) | -0.053 (0.670) | 0.345 (0.004) |
| s10 | 0.779 (<0.001) | 0.703 (<0.001) | 0.367 (<0.001) | 0.551 (<0.001) | 0.773 (<0.001) | 0.695 (<0.001) | 0.353 (<0.001) | 0.591 (<0.001) |
| s11 | --- | --- | --- | --- | 0.887 (<0.001) | 0.825 (<0.001) | 0.521 (<0.001) | 0.545 (<0.001) |
| s12 | 0.857 (<0.001) | 0.767 (<0.001) | 0.482 (<0.001) | 0.389 (<0.001) | 0.865 (<0.001) | 0.767 (<0.001) | 0.453 (<0.001) | 0.388 (<0.001) |
| s14 | 0.813 (<0.001) | 0.440 (<0.001) | -0.159 (0.074) | 0.269 (0.002) | 0.810 (<0.001) | 0.433 (<0.001) | -0.165 (0.064) | 0.253 (0.004) |
| s15 | 0.837 (<0.001) | 0.669 (<0.001) | 0.058 (0.496) | 0.468 (<0.001) | 0.828 (<0.001) | 0.661 (<0.001) | 0.058 (0.491) | 0.448 (<0.001) |
| s17 | 0.652 (<0.001) | 0.400 (<0.001) | -0.201 (0.019) | 0.372 (<0.001) | 0.684 (<0.001) | 0.429 (<0.001) | -0.194 (0.024) | 0.384 (<0.001) |
| Mean Fisher Z | 1.008 (---) | 0.691 (<0.001) | 0.158 (<0.001) | 0.439 (<0.001) | 1.052 (---) | 0.739 (<0.001) | 0.194 (<0.001) | 0.474 (<0.001) |

Strength of correlation indicated by Pearson’s correlation coefficients between ∆CBFv and RGE metrics including bER, ∆PO_2_, ∆PCO_2_ and ToB (n=12). Numbers in brackets next to Pearson’s correlation coefficients indicate p values from individual correlation analyses. The bottom row shows the mean values of Fisher Z scores transformed from Pearson’s correlation coefficients in groups. Numbers in brackets next to mean Fisher Z scores indicate p values in the paired comparisons. The correlation between ∆CBFv and bER was significantly larger than those of the correlation between ∆CBFv and the other respiratory metrics in the paired comparisons (p<0.001). bER is the only parameter that consistently showed significantly high correlation with the ∆CBFv measured in LMCA and RMCA.

**S2 Table B. Correlation between RGE metrics and ∆BOLD in MRI sessions.**

|  | ΔBOLD in LGM | | | | ΔBOLD in RGM | | | |
| --- | --- | --- | --- | --- | --- | --- | --- | --- |
| Subjects | bER | ΔPO_2_ | ΔPCO_2_ | ToB | bER | ΔPO_2_ | ΔPCO_2_ | ToB |
| s1 | 0.674 (<0.001) | 0.558 (<0.001) | 0.044 (0.653) | 0.274 (0.005) | 0.696 (<0.001) | 0.577 (<0.001) | 0.044 (0.656) | 0.304 (0.002) |
| s2 | 0.810 (<0.001) | 0.744 (<0.001) | 0.433 (<0.001) | 0.616 (<0.001) | 0.829 (<0.001) | 0.758 (<0.001) | 0.436 (<0.001) | 0.631 (<0.001) |
| s3 | 0.511 (<0.001) | 0.472 (<0.001) | 0.160 (0.252) | 0.494 (<0.001) | 0.498 (<0.001) | 0.445 (0.001) | 0.109 (0.436) | 0.463 (<0.001) |
| s4 | 0.795 (<0.001) | 0.658 (<0.001) | 0.190 (0.030) | 0.447 (<0.001) | 0.762 (<0.001) | 0.618 (<0.001) | 0.157 (0.075) | 0.435 (<0.001) |
| s5 | 0.352 (0.001) | 0.344 (0.002) | 0.185 (0.101) | 0.410 (<0.001) | 0.579 (<0.001) | 0.555 (<0.001) | 0.352 (0.001) | 0.510 (<0.001) |
| s6 | 0.535 (<0.001) | 0.419 (<0.001) | 0.100 (0.357) | 0.250 (0.020) | 0.576 (<0.001) | 0.452 (<0.001) | 0.118 (0.275) | 0.265 (0.013) |
| s7 | 0.723 (<0.001) | 0.662 (<0.001) | 0.133 (0.204) | 0.490 (<0.001) | 0.786 (<0.001) | 0.742 (<0.001) | 0.234 (0.024) | 0.582 (<0.001) |
| s8 | 0.708 (<0.001) | 0.556 (<0.001) | -0.070 (0.483) | 0.374 (<0.001) | 0.733 (<0.001) | 0.595 (<0.001) | -0.022 (0.826) | 0.398 (<0.001) |
| s9 | 0.673 (<0.001) | 0.582 (<0.001) | 0.233 (0.075) | 0.621 (<0.001) | 0.675 (<0.001) | 0.587 (<0.001) | 0.244 (0.063) | 0.640 (<0.001) |
| s10 | 0.605 (<0.001) | 0.519 (<0.001) | 0.220 (0.026) | 0.469 (<0.001) | 0.735 (<0.001) | 0.659 (<0.001) | 0.366 (<0.001) | 0.576 (<0.001) |
| s11 | 0.175 (0.056) | -0.067 (0.466) | -0.423 (<0.001) | 0.367 (<0.001) | 0.311 (0.001) | 0.069 (0.452) | -0.347 (<0.001) | 0.335 (<0.001) |
| s12 | 0.698 (<0.001) | 0.629 (<0.001) | -0.057 (0.543) | 0.298 (0.001) | 0.672 (<0.001) | 0.601 (<0.001) | -0.063 (0.497) | 0.259 (0.005) |
| s13 | 0.478 (<0.001) | 0.396 (<0.001) | 0.066 (0.528) | 0.298 (0.004) | 0.474 (<0.001) | 0.411 (<0.001) | 0.109 (0.300) | 0.297 (0.004) |
| s14 | 0.614 (<0.001) | 0.551 (<0.001) | 0.047 (0.646) | 0.371 (<0.001) | 0.707 (<0.001) | 0.652 (<0.001) | 0.113 (0.265) | 0.369 (<0.001) |
| s15 | 0.717 (<0.001) | 0.631 (<0.001) | 0.283 (0.005) | 0.375 (<0.001) | 0.414 (<0.001) | 0.355 (<0.001) | 0.146 (0.155) | 0.040 (0.696) |
| s16 | 0.525 (<0.001) | 0.423 (<0.001) | -0.032 (0.775) | 0.304 (0.006) | 0.564 (<0.001) | 0.461 (<0.001) | -0.021 (0.855) | 0.337 (0.002) |
| Mean Fisher Z | 0.727 (---) | 0.579 (<0.001) | 0.096 (<0.001) | 0.436 (<0.001) | 0.767 (---) | 0.620 (<0.001) | 0.127 (<0.001) | 0.441 (<0.001) |
|  | ΔBOLD in LWM | | | | ΔBOLD in RWM | | | |
| Subjects | bER | ΔPO_2_ | ΔPCO_2_ | ToB | bER | ΔPO_2_ | ΔPCO_2_ | ToB |
| s1 | 0.648 (<0.001) | 0.540 (<0.001) | 0.057 (0.560) | 0.268 (0.005) | 0.655 (<0.001) | 0.547 (<0.001) | 0.052 (0.597) | 0.287 (0.003) |
| s2 | 0.792 (<0.001) | 0.740 (<0.001) | 0.472 (<0.001) | 0.626 (<0.001) | 0.806 (<0.001) | 0.741 (<0.001) | 0.451 (<0.001) | 0.621 (<0.001) |
| s3 | 0.543 (<0.001) | 0.480 (<0.001) | 0.096 (0.496) | 0.538 (<0.001) | 0.464 (<0.001) | 0.392 (0.004) | 0.030 (0.833) | 0.419 (0.002) |
| s4 | 0.789 (<0.001) | 0.654 (<0.001) | 0.193 (0.028) | 0.408 (<0.001) | 0.770 (<0.001) | 0.627 (<0.001) | 0.164 (0.062) | 0.428 (<0.001) |
| s5 | 0.403 (<0.001) | 0.394 (<0.001) | 0.237 (0.034) | 0.434 (<0.001) | 0.588 (<0.001) | 0.556 (<0.001) | 0.328 (0.003) | 0.584 (<0.001) |
| s6 | 0.571 (<0.001) | 0.454 (<0.001) | 0.132 (0.223) | 0.262 (0.014) | 0.589 (<0.001) | 0.466 (<0.001) | 0.134 (0.216) | 0.265 (0.013) |
| s7 | 0.548 (<0.001) | 0.497 (<0.001) | 0.081 (0.442) | 0.353 (0.001) | 0.746 (<0.001) | 0.700 (<0.001) | 0.218 (0.036) | 0.515 (<0.001) |
| s8 | 0.702 (<0.001) | 0.558 (<0.001) | -0.053 (0.594) | 0.378 (<0.001) | 0.749 (<0.001) | 0.605 (<0.001) | -0.027 (0.784) | 0.423 (<0.001) |
| s9 | 0.642 (<0.001) | 0.551 (<0.001) | 0.214 (0.103) | 0.591 (<0.001) | 0.679 (<0.001) | 0.585 (<0.001) | 0.232 (0.077) | 0.580 (<0.001) |
| s10 | 0.620 (<0.001) | 0.538 (<0.001) | 0.245 (0.013) | 0.487 (<0.001) | 0.722 (<0.001) | 0.651 (<0.001) | 0.382 (<0.001) | 0.569 (<0.001) |
| s11 | -0.011 (0.902) | -0.184 (0.044) | -0.374 (<0.001) | 0.295 (0.001) | 0.213 (0.019) | 0.043 (0.644) | -0.243 (0.008) | 0.226 (0.013) |
| s12 | 0.634 (<0.001) | 0.571 (<0.001) | -0.043 (0.645) | 0.234 (0.011) | 0.662 (<0.001) | 0.583 (<0.001) | -0.096 (0.303) | 0.253 (0.006) |
| s13 | 0.262 (0.011) | 0.217 (0.037) | 0.039 (0.708) | 0.064 (0.542) | 0.257 (0.013) | 0.241 (0.020) | 0.103 (0.327) | 0.028 (0.793) |
| s14 | 0.581 (<0.001) | 0.569 (<0.001) | 0.174 (0.086) | 0.402 (<0.001) | 0.530 (<0.001) | 0.493 (<0.001) | 0.111 (0.275) | 0.222 (0.027) |
| s15 | 0.742 (<0.001) | 0.656 (<0.001) | 0.308 (0.002) | 0.379 (<0.001) | 0.609 (<0.001) | 0.530 (<0.001) | 0.232 (0.022) | 0.170 (0.097) |
| s16 | 0.500 (<0.001) | 0.405 (<0.001) | -0.025 (0.827) | 0.262 (0.018) | 0.540 (<0.001) | 0.436 (<0.001) | -0.032 (0.775) | 0.310 (0.005) |
| Mean Fisher Z | 0.672 (---) | 0.544 (<0.001) | 0.113 (<0.001) | 0.403 (0.001) | 0.726 (---) | 0.589 (<0.001) | 0.132 (<0.001) | 0.402 (<0.001) |

Strength of correlation indicated by Pearson’s correlation coefficients between ∆BOLD and RGE metrics including bER, ∆PO_2_, ∆PCO_2_ and ToB (n=16). Numbers in brackets next to Pearson’s correlation coefficients indicate p values from individual correlation analyses. The bottom row shows the mean values of Fisher Z scores transformed from Pearson’s correlation coefficients in groups. Numbers in brackets next to mean Fisher Z scores indicate p values in paired comparisons. The correlation between ∆BOLD and bER was significantly larger than those of the correlation between ∆BOLD and the other respiratory metrics in the paired comparisons (p<0.001). bER is the only parameter that consistently showed significantly high correlation with the ∆BOLD measured in LGM, RGM, LWM and RWM
